# Modifiable lifestyle factors are associated with blood‐brain barrier permeability in cognitively normal older adults

**DOI:** 10.1002/alz70861_108722

**Published:** 2025-12-23

**Authors:** Seraphina K. Solders, Emilie T. Reas

**Affiliations:** ^1^ University of California, San Diego, La Jolla, CA USA

## Abstract

**Background:**

Blood‐brain barrier (BBB) breakdown is an increasingly recognized feature of brain aging and dementia. While lifestyle factors, particularly those that influence vascular health, can modify brain aging trajectories, their impact on BBB dysfunction remains understudied. This study examines relationships of modifiable vascular risk factors, including alcohol consumption, smoking, and physical activity, with regional BBB permeability among cognitively normal older adults.

**Method:**

Forty‐six cognitively normal participants (75.65 ± 8.07 years of age; 50% women) from the UC San Diego Alzheimer’s Disease Research Center (n = 41) and San Diego community (n = 5) underwent dynamic contrast‐enhanced magnetic resonance imaging to model BBB permeability and completed a questionnaire assessing lifestyle factors. Average permeability was calculated within regions from the Desikan‐Killiany cortical atlas, FreeSurfer subcortical segmentation, and AtlasTrack white matter (WM) pathways, and averaged across hemispheres. Participants were characterized on alcohol consumption [abstainer, moderate (<= 7 drinks/week), or heavy drinker (>7 drinks/week)]; smoking status (ever or never smoked); lifetime smoking exposure (average cigarettes smoked/day times years smoked); regular engagement in strenuous exercise or labor and exercising at least 3 times/week (yes to both or no); hours/day spent walking; and hours/day spent sitting. Linear regressions predicted regional BBB permeability from each lifestyle measure while controlling for age and sex. Follow‐up models assessed interactions between lifestyle measures and sex.

**Result:**

Heavy drinkers showed elevated parahippocampal cingulum permeability compared to moderate drinkers. Consistent strenuous exercise was associated with higher whole‐brain permeability among women only. More hours spent walking per day were associated with lower global WM permeability. No associations with permeability were found for smoking or sitting.

**Conclusion:**

Alcohol consumption and physical activity were related to BBB permeability in cognitively normal older adults, with sex‐specific patterns warranting further examination. Findings implicate modifiable vascular lifestyle factors in the maintenance of BBB integrity in later life, with potential applications to strategies supporting healthy brain aging.

